# Resistance training to improve type 2 diabetes: working toward a prescription for the future

**DOI:** 10.1186/s12986-017-0173-7

**Published:** 2017-03-02

**Authors:** Dominik H. Pesta, Renata L. S. Goncalves, Anila K. Madiraju, Barbara Strasser, Lauren M. Sparks

**Affiliations:** 1 Department of Sport Science, Medical Section, University of Innsbruck, Fürstenweg 185, Innsbruck, Austria; 2 Department of Visceral, Transplant, and Thoracic Surgery, D. Swarovski Research Laboratory, Medical University of Innsbruck, Innsbruck, Austria; 3 Institute for Clinical Diabetology, German Diabetes Center, Leibniz Institute for Diabetes Research at Heinrich Heine University, Düsseldorf, Germany; 4 German Center for Diabetes Research (DZD e.V.), München-Neuherberg, Germany; 5 Department of Genetics and Complex Diseases and Sabri Ülker Center, Harvard T.H. Chan School of Public Health, 677 Huntington Avenue, Boston, MA 02115 USA; 6 Salk Institute for Biological Studies, 10010N Torrey Pines Rd, La Jolla, CA 92037 USA; 7 Biocenter, Medical University Innsbruck, Innrain 80-82, Innsbruck, Austria; 8 Translational Research Institute for Metabolism and Diabetes, Florida Hospital, 301 E. Princeton Street, Orlando, FL 32804 USA; 9 Sanford Burnham Prebys Medical Discovery Institute, Center for Clinical and Molecular Origins of Disease, Orlando, FL USA

**Keywords:** Resistance training, Type 2 diabetes, Skeletal muscle, Mitochondrial function

## Abstract

The prevalence of type 2 diabetes (T2D) is rapidly increasing, and effective strategies to manage and prevent this disease are urgently needed. Resistance training (RT) promotes health benefits through increased skeletal muscle mass and qualitative adaptations, such as enhanced glucose transport and mitochondrial oxidative capacity. In particular, mitochondrial adaptations triggered by RT provide evidence for this type of exercise as a feasible lifestyle recommendation to combat T2D, a disease typically characterized by altered muscle mitochondrial function. Recently, the synergistic and antagonistic effects of combined training and Metformin use have come into question and warrant more in-depth prospective investigations. In the future, clinical intervention studies should elucidate the mechanisms driving RT-mitigated mitochondrial adaptations in muscle and their link to improvements in glycemic control, cholesterol metabolism and other cardiovascular disease risk factors in individuals with T2D.

## Background

### The significance of resistance training for individuals with type 2 diabetes: moving beyond what we already know

The prevalence of type 2 diabetes (T2D) continues to increase. Within the next 20 years, the number of people affected by this disease is expected to reach almost 600 million worldwide [1]. T2D is accompanied by a host of risk factors including dyslipidemia, hypertension and cardiovascular disease [2], thus putting a severe burden on our global health care systems. Apart from medication, chronic exercise (i.e. systematic training performed repeatedly) is a proven prevention and treatment strategy for individuals with pre-diabetes and T2D [3–5]. Recent reviews and meta-analyses, including the 2016 joint position statement on physical activity and T2D from the American Diabetes Association [6], have highlighted the beneficial effects of chronic endurance training (ET), resistance training (RT) and/or combined (ET + RT) interventions for ameliorating insulin sensitivity and glycemic control in individuals with T2D [7, 8]. Chronic ET alone has a well-established role in enhancing insulin sensitivity via glucose uptake and disposal (reviewed in [9]) and in increasing muscle mitochondrial density and oxidative capacity [10]. A limited number of studies have demonstrated that chronic RT alone enhances glycemic control [11, 12] and muscle substrate metabolism in individuals with T2D [13], yet the underlying mechanisms inducing these health benefits, particularly those related to muscle mitochondrial function, remain to be elucidated.

The present review focuses on the effects of chronic RT on glycemic control, substrate metabolism and the molecular mechanisms that may influence these adaptations in individuals with T2D. We place a special emphasis on skeletal muscle, the interaction between anti-diabetic medication use and training stimulus, and incorporate adipose tissue as another significant target organ for RT-mediated metabolic adaptations in T2D. Since little is known about the independent effects of chronic RT on mitochondrial adaptations in skeletal muscle and adipose tissue of individuals with T2D, we identify gaps in the current literature and raise important questions that future RT-focused trials in individuals with T2D will hopefully address. Improving our understanding of the mechanisms underpinning chronic RT-mitigated metabolic adaptions in T2D will move the scientific community (researchers and clinicians alike) beyond what we already know and toward future investigations focused on molecular determinants of the individual training responses in T2D.

## Chronic resistance training effects on muscle mass, fiber type and glycemic control

### Resistance training-induced gains in muscle mass are not solely responsible for improved muscle substrate metabolism in type 2 diabetes

**Fig. 1:**
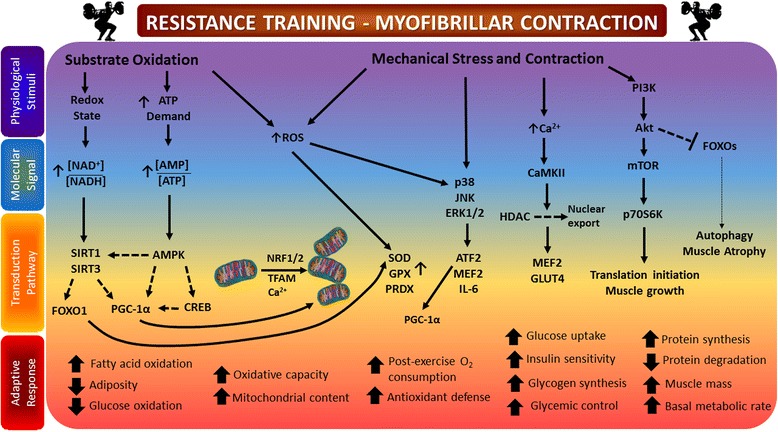
Summarizes the physiological stimuli, triggered by resistance training and the specific molecular signaling events leading to a number of beneficial adaptive responses. These multifactorial benefits induced by resistance training can either be mediated independently of an increase in muscle mass (*e.g.,* increased key insulin signaling proteins resulting in improved insulin action, enhanced post-exercise oxygen consumption resulting in a decrease of adipose tissue mass, increased mitochondrial content positively affecting fatty acid oxidation capacity and improved glucose homeostasis due to augmented rates of glycogen synthesis). The benefits can also be associated with an increase in muscle mass (*e.g.,* improved glycemic control via increased glucose transporter 4 protein expression, increased resting energy expenditure and metabolic demand via increased muscle protein turnover). Increased substrate oxidation during exercise can alter redox state and energy charge, signaling for activation of SIRT family members and AMPK. Downstream activation of PGC-1α and FOXO1 can promote fatty acid oxidation, mitochondrial biogenesis and increased antioxidant effects. ROS signaling during exercise can also promote mitochondrial function and bolster antioxidant defense via SOD, GPX and PRDX. Mechanical stress (*e.g.,* contraction) during exercise triggers calcium signaling that promotes glucose uptake via GLUT4, muscle growth and differentiation via MEF2 and Akt-mTOR, and has a negative effect on the activity of FOXO family members (FOXO1, FOXO3a), minimizing autophagy and muscle atrophy. Please see text for more information. Adapted from [92]. Abbreviations: AMP: Adenosine monophosphate; AMPK: Adenosine monophosphate activated kinase; ATF: activating transcription factor; CaMK: Ca^2+^/calmodulin-dependent protein kinase; CREB: cAMP response element-binding protein; ERK: extracellular signal–regulated kinase; FOXO: Forkhead box protein O; GLUT4: glucose transporter 4﻿﻿; HDAC: Histone deacetylases; IL-6: interleukin 6; JNK: c-Jun N-terminal kinases; mTOR: mammalian target of rapamycin; MEF: myocyte enhancing factor; NAD/H+: Nicotineamide adenine dinucleotide; NRF1/2: nuclear respiratory factor 1/2; p70 S6K: ribosomal protein S6 kinase beta-1; PGC1-α: peroxisome proliferator-activated receptor gamma co-activator 1-alpha; PI3K: phosphatidylinositol-3-kinases; ROS: reactive oxygen species; SIRT: silent mating type information regulation homolog; TFAM: mitochondrial transcription factor A;

Skeletal muscle is responsible for ~80% of insulin-mediated glucose uptake in the postprandial state [14], and uptake is markedly blunted in individuals with T2D [15]. In fact, when compared with lean healthy individuals, skeletal muscle of individuals with T2D exhibits a decreased capacity to oxidize both glucose and fat [16]. Chronic RT increases muscle mass and strength, largely due to induction of muscle hypertrophy and neuromuscular remodeling [17] through the phosphatidylinositol 3 kinase (PI3K)- Akt - mammalian target of rapamycin (mTOR) pathway [18] (Fig. 1). These gains are typically associated with and often surmised to underlie improvements in muscle substrate (glucose and fat) metabolism even in the absence of direct evidence. Recent reports have shown that in addition to increasing strength [12], 9 months of RT enhanced oxidation of both fatty acid- and glycolytic-derived substrates in skeletal muscle of individuals with T2D [13]. The 1.4 kg increase in muscle mass observed was interpreted to be the root cause of these metabolic adaptations, yet many other factors such as improvements in insulin signaling could be responsible for the RT-induced improvements in substrate metabolism and glycemic control [19]. At the molecular level, calmodulin-dependent protein kinase (CaMK) II, a critical sensor for intracellular calcium signaling and muscle remodeling, is activated in an exercise intensity-dependent manner. CaMK II phosphorylates transcription factors such as histone deacetylases (HDACs) [20], which upon phosphorylation are exported from the nucleus leading to activation of transcription factors such as myocyte enhancing factor (MEF) 2 and its target genes [*e.g.,* peroxisome proliferator-activated receptor-gamma coactivator 1 alpha (PGC-1α), glucose transporter protein 4 (GLUT4), thereby improving glycemic control [21] (Fig. 1). Of note, a recent review assessing the different characteristics of ET, RT and combined training interventions and their associations with glycemic control among individuals with T2D concluded that increasing the number of exercise sessions (by volume and frequency) showed greater benefits than either mode or intensity of the training itself [22]. Unfortunately, data regarding the effects of varied intensities and durations of RT on muscle mass are limited. To this point, when expressed per kilogram of body weight, glucose disposal rates are ~40–45% higher in weightlifters—individuals characterized by large amounts of muscle mass—compared to untrained individuals [23]; however, when normalized to muscle mass, glucose disposal rates no longer differ between weightlifters and untrained controls. These results underline the importance of understanding that chronic RT can have separate but equally important impacts on skeletal muscle in terms of strength and substrate utilization, and that while increased muscle mass can contribute to enhanced whole-body glucose-disposal rates, it does not necessarily suggest that the exercise regimen altered the inherent capacity of muscle to more effectively metabolize substrate.

### Metabolic adaptations within skeletal muscle in response to resistance training: how much does fiber type matter?

Type IIx fibers are described as having a glycolytic phenotype, relying on glucose more than fat as a fuel, and facilitating short-duration anaerobic activities. It has been shown that Type IIx fibers are present in a higher proportion in individuals with T2D [4]. Hyperinsulinemia—a hallmark feature of insulin resistance and T2D—can shift muscle fiber type toward a glycolytic phenotype by increasing Type IIx myosin heavy chain gene expression [5]. Physical inactivity and immobilization have similar effects on fiber type shift [21]. Interestingly, first-degree relatives of individuals with T2D have a ~30% higher proportion of Type IIx fibers than individuals without a family history of T2D. Type IIx fiber content was also negatively associated with glucose disposal rates in these same individuals [6]. Paradoxically, elite strength and speed athletes have a high proportion of Type IIx fibers and are metabolically healthy, yet the high proportion of Type IIx fibers observed in individuals with T2D is concomitant with overall blunted substrate oxidation and appears to be less advantageous for these individuals. It is tempting to speculate that the high number of Type IIx fibers in individuals with T2D could be “trained” to utilize fuel more effectively as observed in strength-based athletes. Four to six weeks of moderate intensity RT (at 40–50% of the one-repetition maximum, 1RM) markedly increased skeletal muscle glucose uptake in non-obese individuals with T2D [24], which was largely attributed to a shift in fiber type toward Type IIa fibers. Single fiber analysis revealed that Type IIa fibers were the ones with the highest glucose uptake and GLUT4 content among the Type II fiber population [25, 26]. Type IIa fibers also had a higher capillary density and showed a greater insulin response than Type IIx fibers [27]. Although Type IIa fibers exhibited more marked glycogen depletion during an exercise bout and faster glycogen re-synthesis following the exercise bout [28], it remains to be determined whether altering fiber type distribution benefits individuals with T2D in this respect. It is entirely possible that fiber type composition is irrelevant if the cellular metabolic machinery (*e.g.,* glucose transport, mitochondria, etc.) is dysfunctional. In other words, quantity does not necessarily equal quality. Challenging the idea that switching fiber type confers a metabolic advantage, two independent studies demonstrated that chronic RT-driven improvements in insulin responsiveness and high-density lipoprotein (HDL) levels in individuals with T2D occurred without any changes in fiber type composition [29, 30], a phenomenon routinely observed in healthy individuals [31]. Metabolic adaptations within muscle can therefore occur independently of a change in muscle fiber type composition. It is important to note that direct comparisons of the effects of different durations and intensities of RT on fiber type composition are virtually absent from the literature.

## Chronic resistance training and mitochondrial fitness

### Resistance training effects on muscle mitochondrial function in individuals with type 2 diabetes: how much do we really know?

Perturbations in mitochondrial oxidative capacity play a major role in the development and progression of insulin resistance and T2D [32]. As few as 3 days of high-fat feeding are sufficient to induce insulin resistance and reduce muscle mitochondrial oxidative phosphorylation at the transcriptional level in lean, sedentary individuals [33]. Individuals with T2D have reduced mitochondrial oxidative capacity when expressed per unit of muscle mass compared to healthy individuals; however, when mitochondrial oxidative capacity is normalized to markers of mitochondrial content (*e.g.,* mitochondrial DNA copy number, citrate synthase activity), these differences are insignificant [16, 34]. That being said, disrupted mitochondrial morphology and a 35% reduction in mitochondrial size reported in individuals with T2D are indicative of functional impairment [16].

Aerobic and combined training improve mitochondrial function [35]. Mechanical stress induces activation of the mitogen-activated protein kinase (MAPK) family of proteins (extracellular-regulated kinase (ERK1/2) and p38 MAPK). Activation of p38 MAPK during contraction stimulates activating transcription factor (ATF) 2 and MEF2, which increase PGC-1α expression and improve mitochondrial function in skeletal muscle (Fig. 1) [36]. Some evidence to support a role for chronic RT in increasing muscle mitochondrial oxidative capacity exists in healthy individuals [37–39]; however, similar data in individuals with T2D are limited. RT (alone or in combination with ET) has only recently been recognized as a promising intervention to maintain muscle mitochondrial oxidative capacity [40] and improve the overall metabolic phenotype of individuals with T2D [13, 41]. Discrepancies in the measured outcomes and the details of the training protocols implemented in RT interventions have made it difficult to ascertain the value of implementing chronic RT to improve muscle mitochondrial function in individuals with T2D.

**Fig. 2:**
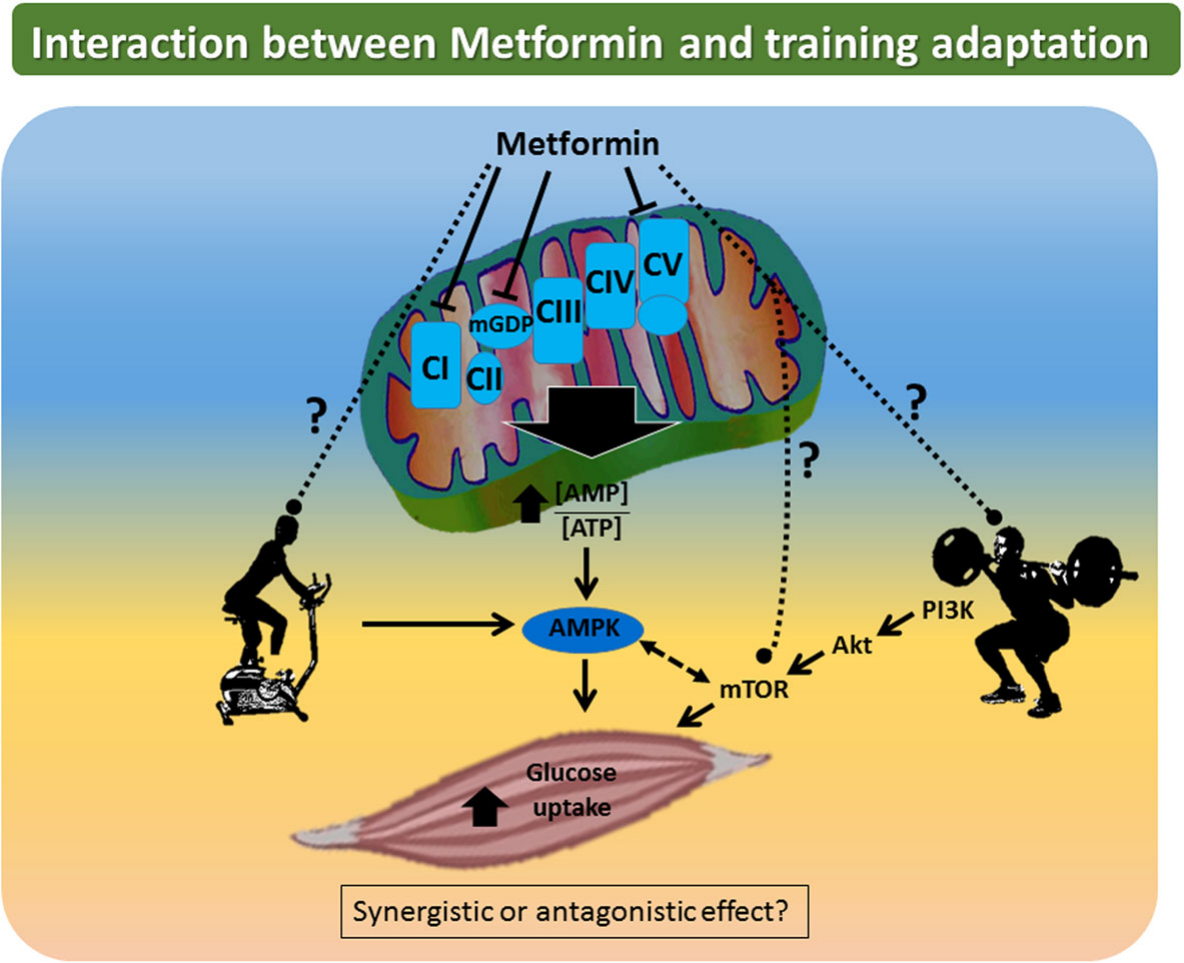
Biguanides such as metformin exert their action via inhibition of complex I, mitochondrial glycerophosphate dehydrogenase and ATP synthase, thereby increasing [AMP]/[ATP] ratio, activating AMPK and increasing insulin sensitivity. ET acts in part through the same pathway, suggesting that these two stimuli could act synergistically. The effect of a RT regimen on individuals with T2D currently taking metformin and other anti-diabetic drugs remains to be determined

Twelve weeks of RT (50–75% of 1RM) twice per week failed to alter PGC-1α protein content and mitochondrial transcription factor A (TFAM) RNA content in individuals with T2D, indicating that this particular duration and/or intensity was not sufficient to induce changes in key regulatory molecules of mitochondrial biogenesis [42]. As mentioned previously, however, quantity does not necessarily equal quality, and the topic of mitochondrial number vs. function is highly debated. According to the classical view of training adaptations, RT signals through the Akt-mTOR-S6K pathway by which it promotes muscle hypertrophy though myofibrillar protein biosynthesis. ET leads to an activation of adenosine monophosphate activated kinase (AMPK) and subsequent activation of PGC-1α, inducing mitochondrial biogenesis [43] (Fig. 2). On the contrary, a recent study demonstrated two-fold higher mRNA expression levels of PGC-1α, PGC-1-related coactivator (PRC) and pyruvate dehydrogenase kinase 4 (PDK4) in a group that performed RT after ET vs. a group that performed ET alone [44]. This suggests that combined (RT + ET) training amplifies the molecular response to ET alone. Decreased energy charge, or an elevated [AMP]/[ATP] ratio in response to contraction, and subsequent AMPK activation possibly mediates changes towards an improved oxidative phenotype by phosphorylating and activating the transcription factor cAMP-response-element binding protein (CREB), which increases PGC-1α transcriptional activity. Exercise-mediated perturbation of redox potential or the [NAD^+^]/[NADH] ratio will also promote the deacetylation activity of silent mating-type information regulation (SIRT) 1 and 3, which further potentiate PGC-1α transcriptional activity [45, 46] (Fig. 1). SIRT1 and SIRT3 activity can lead to deacetylation of FOXO1, allowing nuclear translocation and increased expression of PDK4 that triggers a switch from glucose to lipid oxidation. However, FOXO activity (FOXO1 and FOXO3a) in the muscle has also been implicated in increased autophagy and muscle atrophy via increases in atrogin-1 expression. Interestingly, exercise induction of FOXO activity via effects on redox potential may face inhibitory crosstalk from exercise-mediated activation of the Akt-mTOR pathways, as Akt is known to phosphorylate FOXOs and exclude them from the nucleus [47]. The roles of these seemingly competing pathways in promoting mitochondrial function via enhanced lipid metabolism but maintaining muscle mass remain to be examined in the context of exercise interventions.

One interesting theory on how to improve muscle mitochondrial oxidative capacity in diabetes is via ‘gene shifting’. A recent report suggests that satellite cell activation following chronic RT leads to incorporation of new mitochondrial DNA into mature muscle cells [48]. This phenomenon, known as ‘gene shifting’, may help normalize mitochondrial oxidative capacity in individuals suffering from impaired mitochondrial function by providing undamaged mitochondrial DNA from which new, functional mitochondrial proteins can be synthesized. In support of this hypothesis, a supervised 14-week RT regimen (three times per week at 50–80% of the 1RM) increased cytochrome *c* oxidase (COX) activity, enhanced mitochondrial creatine kinase levels and decreased oxidative DNA damage in elderly men and women [49]. These findings suggest that chronic RT up-regulates selected components of the mitochondrial electron transport system which may convey many of the beneficial effects of this exercise modality [50].

Collectively, these findings suggest that chronic RT is capable of inducing muscle mitochondrial biogenesis and enhancing downstream oxidative capacity. In addition to biogenesis, mitochondria also undergo remodeling (i.e., fusion and fission) to enhance and/or preserve function. RT can act as a trigger to induce mitochondrial fission, followed by recovery-induced fusion and mitochondrial biogenesis. These RT-triggered processes may act as natural selection at the level of the mitochondrion (mito-checkpoint) and eliminate disadvantageous mitochondria, thereby driving adaptive selection of advantageous phenotypic variations [51]. This concept adds another dimension to the current understanding of chronic RT-mitigated improvements in muscle mitochondrial health.

### Resistance training-induced oxidative stress in type 2 diabetes: is defense the best offense?

T2D is characterized by excessive reactive oxygen species (ROS) emission which presumably leads to oxidative stress in different tissues [52, 53]. There are at least 11 sites in muscle mitochondria capable of producing ROS and their rates under different (patho)physiological conditions still need to be determined [54]. Recent estimations assessing *ex vivo* mitochondrial superoxide/H_2_O_2_ production in conditions mimicking physiological states have reported that 0.35% of oxygen consumed at rest is diverted to ROS production, and that this proportion is reduced to 0.03–0.01% during exercise [55]. Given that mitochondria superoxide/H_2_O_2_ production is likely to decrease during moderate exercise, it is important to also consider other cellular reactions and sites as significant mediators of ROS production. Both ET and RT increase oxidant production, which can lead to oxidative stress [56, 57]. ROS can activate c-jun N-terminal kinase (JNK) signaling as evidenced by a recent report demonstrating that infusion of the antioxidant N-acetylcysteine reduced JNK signaling during aerobic exercise training [58] (Fig. 1). Interleukin-6 (IL-6) is associated with a variety of metabolic effects across different organs and its secretion during contraction is JNK-dependent [59], emphasizing the importance of JNK signaling and ROS-mediated JNK activation in facilitating metabolic adaptations to aerobic exercise training [58]. In a comparative study assessing the effects of exhaustive ET vs. RT on ROS production, both training modalities increased oxidative stress; however, lipids were affected by peroxidation only during RT, and proteins were oxidized and formed carbonyls during ET [57]. Training frequency also impacts ROS production and oxidative stress. While a single RT session was sufficient to induce oxidative damage in untrained men [60], 6 months of RT (three times per week at 50–80% of the 1RM) induced whole-body adaptations resulting in decreased training-induced oxidative stress and homocysteine levels [61]. Data on RT-mediated oxidative stress are sparse in individuals with T2D. A recent study reported that 12 months of supervised ET, RT and flexibility training equally reduced oxidative stress in men with T2D [62].

In addition to promoting acute ROS formation [63], aerobic exercise training generally improves muscle antioxidant defense mechanisms [64]. Interestingly, mitochondrial hydrogen peroxide (H_2_O_2_) production is important for muscle differentiation in vitro, and mitochondria-targeted antioxidant treatment with MitoQ and MitoTEMPOL, as well as mitochondria-targeted catalase, blocks this effect [65]. As such, acute ROS formation during RT may also have beneficial effects on muscle differentiation in vivo. Indeed, mitochondrial ROS generation might be involved in initiating mitochondrial biogenesis [66]. Transient ROS “build-up” has been suggested to prime the cellular redox system, which culminates in an improved handling of subsequent pro-oxidant environments. Oxidative stress must therefore be considered in connection with radical scavenging activities and cellular antioxidant defense mechanisms. A 12-week RT intervention (twice per week at 50–75% of the 1RM) not only reduced oxidative stress, but also significantly increased cytosolic and mitochondrial antioxidant proteins [superoxide dismutase-2 (SOD2), glutathione peroxidase-1 (GPX1), peroxiredoxin isoform 5 (PRDX5), heat-shock-protein-70 (HSP70)] in muscle of individuals with T2D [67].

Chronic RT effects on ROS production in human muscle are relatively unexplored. Due to the absence of reliable techniques to precisely measure ROS production in vivo, fundamental questions regarding the sources of ROS and the effects of training modalities on ROS signaling remain unanswered. As such, more studies are required to elucidate how ROS affects the hypertrophic and adaptive responses of human muscle to chronic RT, especially in T2D. Unraveling these pathways could lead to a better understanding of the potential therapeutic role of antioxidant supplements in treating metabolic impairments. Furthermore, it is important to consider the interaction of medication or supplement use with the antioxidant defense system (Fig. 2). A recent review explored the novel hypothesis that attenuation of oxidative stress from exercise training by these exogenous compounds blunts beneficial metabolic adaptations [68].

## Chronic resistance training and medication use

### Metformin and exercise training in diabetes: the good, the bad and the unknown

In recent years, the combined effects of metformin treatment and training regimens on metabolic outcomes have gained attention, the majority of these studies conducted in healthy and/or pre-diabetic populations [69–73] (Fig. 2). Metformin is the most widely prescribed first-line oral anti-diabetic drug recommended by the American Diabetes Association (ADA). The exact molecular mechanism of action underlying this drug’s physiological benefits remains mysterious, although it is often prescribed together with a regular exercise training program as part of a lifestyle intervention. Metformin is known to have pleiotropic effects and multiple potential target pathways. One possible site of metformin action is inhibition of mitochondrial respiratory system complex I, increasing the [AMP]/[ATP] ratio (much like muscle contraction does) and activating AMPK. AMPK phosphorylation of multiple downstream effectors can divert cellular metabolism towards restoration of energy homeostasis [74], thereby improving insulin sensitivity (Fig. 2). ET-mediated increases in insulin sensitivity also act in part through AMPK activation [75], suggesting that exercise training and metformin could have additive effects. In support, a recent randomized, controlled chronic exercise training (ET, RT, ET + RT) intervention (22 weeks) in individuals with T2D demonstrated that compared with controls, ET led to a significant reduction in HbA1c in metformin users (-0.57%) but not in untreated individuals (-0.17%). However, metformin did not have any effect on improvements in indicators of aerobic fitness, strength, body weight or waist circumference [76]. Importantly, this effect was only observed with ET and ET + RT. The RT only group did not achieve significant reductions in HbA1c (with or without metformin) compared with the control group.

Alternatively, metformin was also documented to have opposing effects on fat oxidation during and following a single bout of ET in healthy individuals [77], and a recent report demonstrated that metformin blunted the full effect of 12 weeks of combined ET + RT on insulin sensitivity by 25–30% in glucose intolerant individuals [72]. This same investigation demonstrated that metformin use blunted some of the beneficial effects of the combined training related to a reduction in cardiovascular disease risk such as systolic blood pressure or high sensitivity C-reactive protein (hs-CRP) [70]. These findings conflict with an earlier investigation in insulin-resistant individuals in which two to three weeks of metformin treatment blunted the effects of a single bout of ET on insulin sensitivity [71]. The above studies suggest some positive and negative interactions between metformin use and adaptations to both single and repeated bouts of ET and combined ET + RT on glycemic control. Taken together, these data suggest that when exercise training is implemented in a population of current metformin users, which is commonplace in a clinical setting, it is important to consider how use of this medication may affect an individual’s training response in terms of glycemic control.

Biguanides such as metformin have also been recently identified as a new class of mitochondrial glycerophosphate dehydrogenase (mGPD) [78] and ATP synthase inhibitors [79]. Inhibition of ATP synthase may affect energetics in skeletal muscle and recovery of energy charge during exercise. Inhibition of mGPD by metformin leading to decreased cytosolic [NAD^+^]/[NADH] may alter activity of SIRT1, decreasing deacetylation of PGC-1α and reversing potential positive impact of exercise on mitochondrial biogenesis. Also relevant is the recent finding that metformin’s effect as a cancer therapeutic is partially mediated by inhibition of mTOR, raising the question of whether metformin may influence the positive effects of Akt-mTOR pathway activation on muscle mass [20]. It remains to be determined how a chronic RT regimen would impact individuals with T2D currently taking metformin and other anti-diabetic drugs (Fig. 2). More research on the detailed effects of metformin on adaptations to ET and RT, especially in the context of varying intensities and duration, is warranted. Furthermore, the significance of metformin effects directly in the muscle at prescribed doses requires attention, as it is thought that metformin primarily targets liver metabolism. It is possible that metformin-mediated amelioration of whole-body glucose homeostasis via inhibition of uncontrolled liver gluconeogenesis may be enough to restore muscle insulin sensitivity and therefore promote beneficial effects of exercise training in the muscle. Based on current evidence, we speculate that in the muscle, anti-diabetic drugs such as metformin – due to inhibition of complex I of the electron transport system and subsequent AMPK activation – may interfere with adaptations to ET to a greater extent than RT, which recruits a different signaling cascade than AMPK. This could possibly establish RT as a reasonable training modality for a cohort taking metformin, particularly those individuals with T2D.

## Chronic resistance training and adipose tissue

### Adipose tissue is an important (and understudied) target organ of resistance training in diabetes

Studies of exercise training, chronic RT in particular, on white adipose tissue (WAT) remodeling in humans are sparse. WAT secretes two major pro-inflammatory cytokines: interleukin-6 (IL-6) and tumor necrosis factor-alpha (TNF-α). Obesity is characterized by a state of chronic low-grade inflammation, which is implicated in the pathogenesis of T2D [80], and circulating levels of both IL-6 and TNF-α are inversely related to glycemic control and insulin sensitivity [81]. Modulation of low-grade inflammation by 12 weeks of ET (40 minutes at 60–80% of the maximal heart rate) and RT (three times per week at 60–80% of the 1RM) was investigated in obese individuals with T2D. Although ET was more effective in reducing adipocytokines in response to the chronic training, RT also significantly reduced circulating levels of TNF-α and IL-6 [82]. RT for 16 weeks (three times per week at 60–85% of the 1RM) in obese adolescents significantly reduced IL-6 and TNF-α plasma levels, and changes in muscle strength were directly related to changes in pro-inflammatory cytokines [83]. These data suggest that chronic RT induces a signaling pathway that alleviates systemic inflammation. In another study, 12 weeks of supervised RT (3 days per week) in individuals with T2D decreased plasma levels of hs-CRP, a non-specific marker of inflammation, and increased the beneficial adipokine Visfatin [84]. According to some studies [85, 86] excess post-exercise oxygen consumption is higher after RT than after ET. This phase is characterized by utilization of fat as a fuel, which could benefit weight loss [87]. Although data are limited, collectively, these studies highlight the systemic anti-inflammatory effects of RT (presumably via WAT) and the potential of chronic RT to improve body composition and alleviate chronic low-grade inflammation associated with obesity and T2D. How chronic RT affects WAT function (*e.g.,* fibrosis, angiogenesis, browning, etc.) and signaling pathways within the organ itself are areas of research that require greater attention and could shed light on exercise training targets that elicit a positive metabolic response in individuals with T2D.

## Closing remarks

### Future directions – toward personalized medicine?

Due to the large inherent variability of responses to the same RT program, general recommendations are less than ideal. The future of T2D research is moving in the direction of personalized medicine. Current research in this area continues to discover signaling pathways that differ even amongst the most homogenous groups of individuals, and these differences ultimately lead to variations in their physiological responses to medications and treatments. It is imperative that we exploit these inter-individual responses following exercise training interventions in individuals with T2D to maximize each person’s beneficial adaptation to a training program. A clear distinction of the different types of RT will be necessary for the implementation of exercise training as a feasible lifestyle modification in light of current and future progression toward personalized medicine. Different RT programs (with varying intensities) will lead to diverse results and metabolic outcomes, and this cause-effect relationship must be clearly established for RT in order to maximize the benefits for individuals with T2D. In this effort, more research comparing supervised ET interventions with RT interventions of varying intensities, durations and volumes including long-term training studies using different modes of periodization, is required. This will further elucidate exercise-mediated effects on whole-body metabolism and muscle mitochondrial function, specifically in individuals with T2D. In addition, the effects of RT on other major target organs, such as WAT, require deeper investigation.

Another question to be addressed is whether the underlying mechanisms by which chronic RT improves muscle glucose regulation are the same as those utilized by chronic ET. It is necessary to determine which pathways are recruited by each type of exercise training, once again due to the possibility that one training method may impart greater benefit to certain individuals than to others as a result of their genetic makeup, physiology, and current medication use. Differences in sex, age and ethnicity likely contribute to the distinct outcomes of several studies looking at the metabolic effects of endurance and resistance training, even when the exercise protocol itself remains the same. While some studies report beneficial effects of RT on diverse metabolic parameters [19, 88, 89], others do not [90, 91]. It is therefore critical to report the training load, duration and relative intensity when comparing different groups of individuals. Addressing why discrepancies exist in outcomes from studies where individuals trained at similar intensities and duration may hold the key to effectively incorporate exercise training in the treatment of metabolic disease on a personalized level.

## Conclusions

RT is a promising strategy to promote overall metabolic health in individuals with T2D via improvements in muscle mitochondrial performance and increases in muscle mass that may positively impact insulin responsiveness and glucose control. Multifactorial events contribute to the beneficial adaptations elicited by chronic RT (Fig. 1). A consistent regimen of RT at moderate intensity elicits the most beneficial metabolic responses—in terms of HbA1c and insulin sensitivity—in individuals with T2D. Current exercise recommendations should ideally be a synthesis of gains in muscle mass (higher intensity RT) and mitochondrial oxidative capacity (lower intensity RT or ET), as well as aim to reduce circulating pro-inflammatory cytokines (targeting WAT), in order to fully exploit the salutary effects of training on overall metabolic health. This can most efficiently be achieved by utilizing combined (RT and ET) training regimes. Another important consideration for current diabetes treatment guidelines is the use of metformin in combination with exercise training as the question of synergism vs. antagonism remains unanswered (Fig. 2). Combinations of lifestyle interventions (*e.g.,* such as medication use, caloric restriction and weight loss) may offer additional benefits and additive effects to a RT regime.
